# Control of the Adaptive Immune Response by Tumor Vasculature

**DOI:** 10.3389/fonc.2014.00061

**Published:** 2014-03-28

**Authors:** Laetitia Mauge, Magali Terme, Eric Tartour, Dominique Helley

**Affiliations:** ^1^INSERM U970, PARCC (Paris Cardiovascular Research Center), Université Paris-Descartes, Sorbonne Paris Cité, Paris, France; ^2^Service d’Hématologie Biologique, Hôpital Européen Georges Pompidou, Paris, France; ^3^Service d’Immunologie Biologique, Hôpital Européen Georges Pompidou, Paris, France

**Keywords:** endothelial cell, adaptive immunity, tumor, lymphocyte infiltration, immunological synapse

## Abstract

The endothelium is nowadays described as an entire organ that regulates various processes: vascular tone, coagulation, inflammation, and immune cell trafficking, depending on the vascular site and its specific microenvironment as well as on endothelial cell-intrinsic mechanisms like epigenetic changes. In this review, we will focus on the control of the adaptive immune response by the tumor vasculature. In physiological conditions, the endothelium acts as a barrier regulating cell trafficking by specific expression of adhesion molecules enabling adhesion of immune cells on the vessel, and subsequent extravasation. This process is also dependent on chemokine and integrin expression, and on the type of junctions defining the permeability of the endothelium. Endothelial cells can also regulate immune cell activation. In fact, the endothelial layer can constitute immunological synapses due to its close interactions with immune cells, and the delivery of co-stimulatory or co-inhibitory signals. In tumor conditions, the vasculature is characterized by an abnormal vessel structure and permeability, and by a specific phenotype of endothelial cells. All these abnormalities lead to a modulation of intra-tumoral immune responses and contribute to the development of intra-tumoral immunosuppression, which is a major mechanism for promoting the development, progression, and treatment resistance of tumors. The in-depth analysis of these various abnormalities will help defining novel targets for the development of anti-tumoral treatments. Furthermore, eventual changes of the endothelial cell phenotype identified by plasma biomarkers could secondarily be selected to monitor treatment efficacy.

## Introduction

The effects of immunity on tumoral angiogenesis are well-known for several years, but the description of a modulation of immunity by pro-angiogenic molecules, like vascular endothelial growth factor (VEGF), is more recent (1). The use of anti-angiogenic molecules has confirmed this relation, as anti-angiogenic treatments can decrease the infiltration of T-regulatory lymphocytes (Tregs) and myeloid-derived suppressor cells (MDSCs) and lead to a Th1-immunity profile (2, 3). Endothelium itself is implied in the regulation of inflammation and growing evidence may suppose a contribution of tumor endothelium in the development of intra-tumoral immunosuppression. The link between angiogenesis and immunity is of great interest as immunosuppression is considered as the main mechanism implied in the escape of tumor from anti-tumor immunity and also to some conventional cancer therapies (chemotherapy, anti-angiogenic molecules…) (4, 5).

### Intra-tumoral immunosuppression

To allow its development, the tumor has to promote neoangiogenesis and escape the immune system, which constitutes major hallmarks of cancer (6). To suppress immune functions, tumors can inhibit different stages of the immune response induction. First, the tumor environment can disrupt dendritic cell (DC) function of antigen-presenting cells to limit the generation of tumor reactive T cells (7), via transforming growth factor (TGF-β), interleukin (IL)-10, macrophage colony-stimulating factor (M-CSF), IL-6, hypoxia, and lactic acid. VEGF blocks the maturation of DCs. Immature DCs express intermediate amounts of major histocompatibility complex (MHC) class I and II and co-stimulatory molecules, high amounts of co-inhibitory molecules, and immunosuppressive cytokines, thus inducing anergy of effector T cells and expansion of Treg (7). Secondly, homing of T cells and their engraftment can be impaired in tumors, via the modulation of T cell attracting chemokines and the induction of a prohibitive tumor vasculature (7). Then, tumors can promote immunosuppressive cells’ induction and infiltration, like Treg and MDSC (8). Tregs are comprised of natural Tregs, which are thymically derived cells of FoxP3 lineage, and inducible Tregs, which upregulate FoxP3 expression and are derived in the periphery from naïve CD4+ T-cell precursors under tolerogenic conditions. In cancer, Tregs can produce suppressive cytokines and secreted molecules, induce T-cell cytolysis, and modulate the interactions with DCs toward immunosuppression (9). MDSCs are a heterogeneous population of activated immature myeloid cells that is characterized by an increased production of potent suppressors of various T-cell functions, like reactive oxygen and nitrogen species, by an upregulation of the expression of arginase and inducible nitric oxide synthase (10). One mechanism to promote these immunosuppressive populations is the secretion by tumor cells of immunosuppressive products such as prostaglandin E2 (PGE2), VEGF, IL-10, and TGF-β that favor Treg induction and expansion (9). Recent studies have precised the role of VEGF, which directly contributes to the expansion of Treg (11), the recruitment of MDSCs, and the inhibition of DC maturation (12). The secretion of chemokines, like CCL22 and CCL28, by tumor cells and the microenvironment contribute to the recruitment of immunosuppressive cells in the tumor (13–16). Finally, tumors can decrease their MHC I molecule expression, not to be recognized, or express molecules that induce T-cell cytolysis (FasL, TRAIL) and co-inhibitory molecules (programed death ligand 1-PD-L1-, PD-L2, B7-H4) (17). They can also induce tolerance by promoting the expression of inhibitor co-stimulatory molecules by T lymphocytes, like PD-1, T-cell immunglobulin and mucin domain-containing molecule 3 (TIM-3), or cytotoxic T lymphocyte antigen 4 (CTLA-4) (7).

### Endothelium and immunity

Since several years, endothelium has been considered as a whole organ with a unique situation, as it communicates both with the circulating compartment and the tissue (18). Endothelium exerts many functions from the regulation of vascular tone to that of inflammation and hemostasis. But endothelial cells (ECs) display profound heterogeneity depending on their anatomic position within the vascular tree. This position is defined by their embryological origin (19) and exposes endothelial cells to different microenvironments (19). The biomechanical parameters, like shear stress and blood flow, associated to biochemical parameters, like oxygen content and pH of the blood, chemokines, hormones, components of the extracellular matrix, regulate the phenotype of the vasculature (20). Endothelial phenotype is thus also influenced by environment modifications. Acquisition of new capacities by resting endothelial cells under these modifications is referred to as endothelial activation. Resting endothelial cells maintain blood fluidity, regulate blood flow, control vessel wall permeability, and quiesce circulating leukocytes (21). But activated endothelium can be pro-thrombotic, constrictive, and pro-inflammatory in order to manage a pathological situation.

Immune modulation by the endothelium is favored by the unique position of ECs, exposing them to T cells during extravasation from the circulation into the tissue or the tumor. Several steps are implied in the regulation of the immune response by the endothelium. Immune cells must firstly adhere and cross the vascular barrier before being effective at the site of inflammation. Moreover, it has been demonstrated that activated ECs could present antigens to lymphocytes. Depending on the presence of various sets of co-stimulatory signals, they could activate memory cells or anergize naïve T cells (21). Under activation, they could also produce vasodilators, chemokines, and matricial proteins favoring the recruitment of inflammatory cells. Variations in the expression and synthesis of diverse molecules can lead to modifications in the regulation of leukocyte trafficking and lymphocyte activation. Acute inflammation results from type I activation responses, mediated by ligands of heterotrimeric G-protein coupled receptors, or from type II activation by inflammation cytokines (22). Then, adaptive immunity modulates endothelial cell phenotype to polarize the inflammatory reaction. Chronic inflammation usually induces angiogenesis and the formation of tertiary lymphoid organ (22). In a tumoral context, chronic inflammation promotes every step of tumorigenesis, from initiation through tumor promotion, all the way to metastatic progression (23). The presence of tertiary lymphoid organ has been described in several tumor types as a good prognosis factor (24–26), but the tumor develops several mechanisms to limit the immune reaction as described in Section “Intra-tumoral Immunosuppression.”

Tumor vasculature has been described as abnormal, with tortuous, leaky, and immature vessels that are finally less functional (27). Characteristic phenotypes of tumor endothelium have also been described and some studies identified markers specific of tumor endothelial cells (TECs) (28–33). Tumor vasculature abnormalities results from the imbalance between pro- and anti-angiogenic factors in the specific microenvironment to which it is exposed, usually described as hypoxic, rich in VEGF and other growth factors, with irregular blood flow (27). However, tumor microenvironments are heterogeneous among tumor types and the stages of development, and different mechanisms could be implied in the regulation of the immuno-modulating phenotype of TECs. In this review, we will focus on the changes in tumor endothelium phenotype that have been or could be implicated in the suppression of the intra-tumoral adaptive immunity and the mechanisms controlling them when they have been described.

## Control of Intra-Tumoral Lymphocyte Infiltration by Endothelial Cells

**Figure 1:**
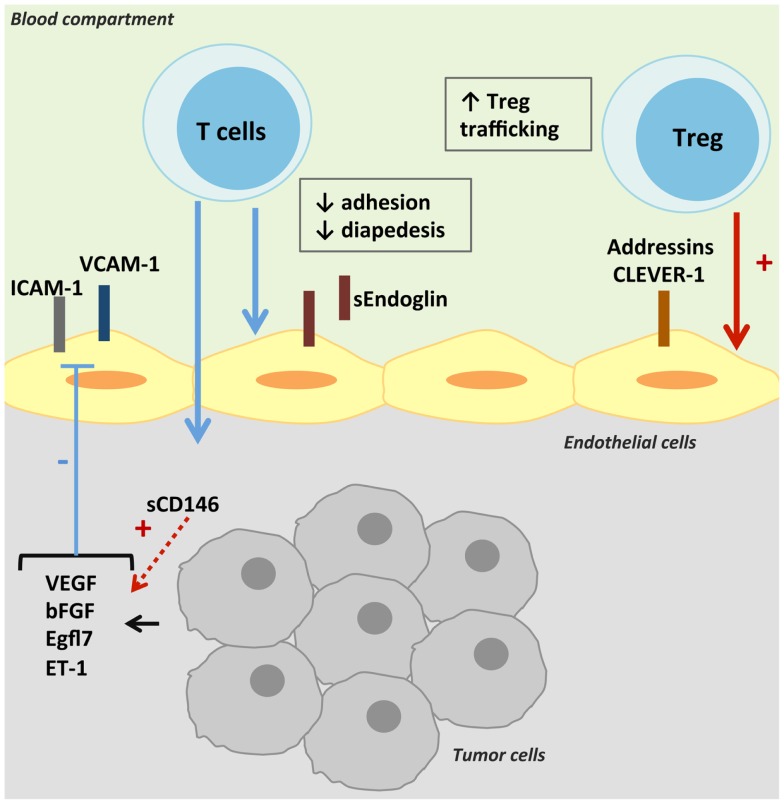
**Control of intra-tumoral immune cell trafficking by tumor endothelial cells**. T-cell trafficking is reduced in tumors despite the secretion of TNF-α. Pro-angiogenic factors present in the tumor microenvironment decrease the expression and/or gathering of adhesion molecules on TECs, and thus T-cell intra-tumoral infiltration. TECs can also express molecules specific of Treg adhesion and transmigration, thus promoting anti-tumoral immune suppression.

Briefly, rolling of leukocytes occurs by the interaction between the selectins expressed by leukocytes and their ligands on ECs (21). Then, leukocyte integrin activation permits its binding to its adhesion ligand expressed by the endothelium. Finally, leukocyte infiltration depends on the chemokine gradient and the type of junctions between ECs (21). As observed in physiological and pathological conditions, ECs can differentially express leukocyte adhesion molecules. Resting ECs lack surface molecules that can initiate tethering, under the control of nitric oxide (NO) and other anti-inflammatory molecules secreted by ECs (34). Maintenance of adherens and of tight junctions in resting EC may further restrict transendothelial leukocyte passage (34). Under different mechanisms – cell interaction or cytokine effect – the expression of adhesion molecules and the synthesis of chemokines are upregulated (34, 35). The prohibitive nature of the tumor endothelium can be mediated by the type and levels of adhesion molecules expressed and must be maintained by local soluble tumor factors (36). The mechanisms reviewed in this part are presented in Figure 1.

### Decrease of cell adhesion molecules

In human solid tumors, a decreased expression of cell adhesion molecules (CAMs) on the vasculature has been described, impairing the development of an efficient leukocyte infiltration in tumors. The high level of tumor necrosis factor α (TNF-α) found in tumors, like in inflammatory responses, should upregulate CAM expression on ECs. But tumor microenvironment may deliver other products that exert a negative regulation. Indeed, Griffioen et al. observed a decrease of TEC activation under exposure to pro-inflammatory cytokines, like TNF-α, that could be attributed to angiogenic factors highly expressed in tumors, basic fibroblast growth factor (b-FGF), or VEGF. The slightest activation was characterized by a limited increase of intercellular cell adhesion molecule-1 (ICAM-1) and 2 (ICAM-2), vascular cell adhesion molecule-1 (VCAM-1), and E-selectin expression, and a decreased *in vitro* adhesion of leukocytes (37). The NO pathway seems to be implicated in the effect of VEGF on lymphocyte–endothelium interactions. In resting ECs, a basal production of NO actively inhibits leukocyte adhesion and activation, by reducing the expression of important adhesion molecules like P-selectin, ICAM-1, and VCAM-1, and maintaining adherens and tight junctions (38). Conversely, NO antagonists can abrogate the deregulation of CAMs induced by VEGF or endothelin-1 (ET-1) and restore T-cell adhesion (39, 40). Bouzin et al. demonstrated that VEGF did not influence the abundance of CAMs at the cell surface, but decreased the expression of caveolin-1 via stimulation of NO pathway, leading to a defect in ICAM-1 and VCAM-1 clustering at the EC surface (40), which is implicated in transendothelial migration (41).

Other molecules have been shown to decrease CAM expression, like epidermal growth factor-like domain 7 (Egfl7) and endothelin-1. *Egfl7* also known as *Vascular Endothelial–statin* gene is mostly expressed in ECs and endothelial progenitors during embryonic and neonatal development. Egfl7 regulates vascular integrity and smooth muscle cell migration (42). An upregulation of egfl7 expression has been observed in ECs during vascular remodeling, such as in reproductive organs during pregnancy, in regenerating endothelium following arterial injury, in atherosclerotic plaques, and in growing tumors (42, 43). Its expression was thought specific of ECs but has also been detected in tumor cells (44). In tumors, levels of Egfl7 are correlated with markers of metastasis and with poor prognosis (45). In glioma, Egfl7 levels correlate with tumor grade (46). Egfl7 can promote tumor growth by repressing ICAM-1 and VCAM-1 expression, then limiting immune cell infiltration, as observed in breast and lung carcinoma murine models (44). Endothelins and their receptors are over-expressed in high-grade glioma, colon cancer, and breast cancer in humans (44). ET-1 is produced by endothelial cells and has a strong vasoconstrictive effect on smooth muscle cells via the endothelin A receptor. But ET-1 induces vasodilatation when binding on the endothelin B receptor [ET(B)R] expressed by the endothelium via induction of nitric oxide secretion. Endothelins also regulate multiple aspects of angiogenesis (47). Indeed, a stimulatory interaction between VEGF and ET-1 has been described on each gene expression (48). ET-1 synthesis is induced by hypoxia, shear stress, and ischemia (21) and ET-1 can promote VEGF secretion by tumor cells (49–51). An overexpression of ET(B)R by TECs has been associated with a decreased ICAM-1 expression and an absence of tumor-infiltrating lymphocytes (TILs), and identified as a poor prognosis marker (39). As for VEGF, NO antagonists can abrogate the deregulation of CAMs induced by ET-1 and restore T-cell adhesion (42).

### Inhibition by soluble cell adhesion molecules

A competitive binding of soluble adhesion molecules could also be hypothesized to explain the decrease in leukocyte infiltration. Endoglin, an auxiliary receptor of the TGF-β family of proteins essential for angiogenesis, is predominantly expressed in vascular ECs (52). Endoglin haploinsufficiency is responsible for hereditary hemorrhagic telangiectasia type 1, characterized by telangiectases and arteriovenous malformations (53). A high expression of endoglin would be a potent marker of solid tumor vasculature (52). Recently, endoglin has been involved in leukocyte trafficking by interacting with α_5_β_1_ integrin (VLA-5) expressed on leukocytes (54). In the same study, an inhibition of leukocyte adhesion by soluble endoglin was observed. The soluble form of endoglin could be involved in the suppression of anti-tumor immune response as increased levels in serum and plasma from cancer patients have been reported as a marker of poor prognosis (55, 56).

CD146, also known as melanoma cell adhesion molecule (MCAM) or S-Endo-1 antigen, is a component of the endothelial junction involved in the control of cell cohesion and tumor angiogenesis (57). CD146 is expressed by ECs but also by several types of cancer cells, smooth muscle cells, follicular DCs and has been described on activated lymphocytes and perivascular cells. As for endoglin, a soluble form of CD146 has been described, with chemotactic and angiogenic properties (58). The role of CD146 in tumors needs to be further defined. Indeed, both membrane and soluble forms of CD146 are involved in monocyte and lymphocyte trafficking (59, 60). However, soluble CD146 can induce VEGFR2 and VEGF expression in a model of hind-limb ischemia, thus promoting angiogenesis (58). The angiogenic role of membrane and soluble CD146 seems predominant, as AA98 antibody directed against CD146 can inhibit tumor growth in xenograft mice (61). Poor data are available on the level of soluble CD146 in tumors, which could be implied in a decrease of lymphocyte infiltration in tumors, either directly or throughout VEGF expression induction.

### Expression of selective cell adhesion molecules

Another way to decrease anti-tumoral immunity is the expression of adhesion molecules favoring specific intra-tumoral infiltration of immunosuppressive populations. For example, tumor-associated vessels in hepatocellular carcinoma present an increased level of common lymphatic endothelial and vascular endothelial receptor-1 (CLEVER-1). This recycling and intracellular trafficking receptor has been implicated preferentially in transendothelial migration of CD4+ FoxP3+ regulatory T cells (62). Its expression seems to be organ-specific and is enhanced by hepatocyte growth factor but not by classical pro-inflammatory cytokines. A selective recruitment of Treg has been observed in tumor tissue of human pancreatic carcinoma due to an increased expression of a broad variety of T-cell transmigration-relevant addressins on tumor endothelium: E-selectin, ICAM-1 and -2, MAdCAM-1 (mucosal vascular addressin cell adhesion molecule-1), VCAM-1, or CD166 (63). Addressin expression on TECs has also been described to be modulated by tumor-derived factors and may vary depending on tumor microenvironment.

## Control of Anti-Tumoral Lymphocyte Reactivity

**Figure 2:**
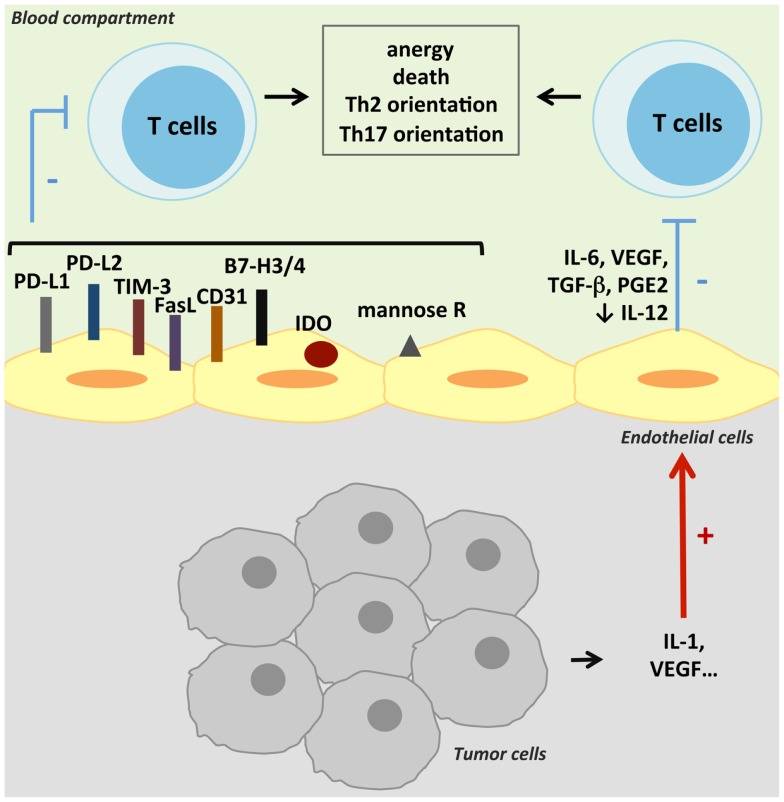
**Suppression of anti-tumoral T-cell activity**. TECs can suppress anti-tumoral adaptive immunity by inducing T-cell anergy or death. This suppression can result either from a cell contact negative signal as TECs can express co-inhibitory molecules, or from inhibitory cytokines secreted by TECs. Various other mechanisms can lead to T-cell activity inhibition, like antigen presentation by the mannose receptor (mannose R) or the expression of IDO.

Two signals are required for induction of cell proliferation and cytokine production in resting T cells. Occupancy of the T-cell receptor (TCR) by antigen presented by the MHC delivers the first signal to the T cell, while the second signal is provided by the interaction with co-stimulatory ligands on APCs. Formation of an immune synapse may serve to stabilize adhesion and extend the duration of bidirectional signaling between the APC and the T cell. Immune synapses are constituted by a concentration of adhesion molecules at the edge, while TCR/MHC complexes and co-stimulatory molecules are grouped in the center. A modulation in MHC II, co-stimulatory/co-inhibitory molecule, or cytokine expression by TECs could participate to the tumor-induced immunosuppression. The major mechanisms reviewed in this part are presented in Figure 2. Some of them have been described in tumors; others exist in physiologic or pathologic conditions and could participate in tumoral immunotolerance.

### Antigen presentation

Antigen-presenting cell function of TECs is defined by their capacity to present exogenous antigens by class II MHC. The antigen uptake, processing, and presentation are regulated by the microenvironment as pro-inflammatory cytokines like TNF-α and interferon-γ (IFN-γ) can upregulate the expression of class II MHC and accessory molecules (34). The ability of ECs to enhance memory T-cell activation is well-established, and has been attributed to the presence of both MHC and co-stimulatory molecules on ECs (64, 65). Adhesion molecules are involved in the formation of immunological synapses, which need the same docking structures than required for transendothelial migration (34). Modulation of the expression of adhesion molecules will then influence the regulation of lymphocyte reactivity by TECs. The consequence of antigen presentation by TECs will finally also depend on the type of co-stimulatory signals interacting with T cells.

Other mechanisms of exogenous antigen intake have been described in tumors, leading either to favor or to limit anti-tumor immunity. Indeed, tumor antigen peptide transfer in TECs has been observed *in vitro* (66). Formation of gap junctions between melanoma cells and ECs led to a transfer of tumor peptides and to a specific killing of tumor-surrounding cells by autologous cytotoxic T lymphocytes (66). This mechanism should favor anti-tumor immunity but co-stimulatory signals may be modulated upon tumor microenvironment. Conversely, antigen uptake, processing, and presentation to T cells by liver sinusoidal ECs via the mannose receptor (67) have been suggested to limit local immune response in the liver (68). In a model of colon carcinoma, tumor cell interaction with liver sinusoidal endothelium through ICAM-1 led to a cyclo-oxygenase 2-dependent IL-1 production, which upregulated the expression of mannose receptor on TECs and decreased anti-tumor activity of interacting lymphocytes (69).

### Co-stimulatory signals

The costimulation necessary to activate resting T cells may be mediated by three types of molecules: signaling molecules (true co-stimulators), adhesion molecules, and activating cytokines. Tumor microenvironment controls T-lymphocyte activation by several mechanisms, among them the inhibition of DC maturation. Indeed, immature DCs express intermediate amounts of MHC class I and II and co-stimulatory molecules, high amounts of co-inhibitory molecules, and immunosuppressive cytokines, thus inducing anergy of effector T cells and expansion of Treg. The tumor microenvironment also induces the expression of co-inhibitory molecules on T lymphocytes that interact with their ligands expressed on tumor cells. Interestingly, TECs can also express co-inhibitory molecules and produce immunosuppressive molecules, and thus may participate to anergize T cells in the tumor microenvironment.

#### Surface co-stimulatory molecules

Human ECs cultured from different vessel sources express numerous co-stimulatory molecules, including LFA-3, OX40-L, 4-1BB-L (CD137), ICOS-L, and glucocorticoid-induced TNF-receptor-related protein (GITR-L) (70). Among the activating co-stimulatory signals, human endothelial costimulation of T cells can be attributed in large part to LFA-3, which binds to CD2 on human T cells (71). Human ECs constitutively do not generally express either CD80 or CD86, with some possible exceptions (34). They express CD40, often described as a co-stimulator of T-cell activation, although it is uncertain if engagement of the CD40 ligand on T cells actually delivers a co-stimulatory signal to the T cell (70).

Among co-inhibitory molecules, ECs can express PD-L1, which bind to PD-1 on activated lymphocytes and down-regulate T-cell activation (72). Like many other co-signaling molecules, PD-L expression is upregulated by pro-inflammatory cytokines like IFN-γ (73). Conversely, signaling by PD-L pathway led to inhibition of IFN-γ secretion and CD8 cytolytic activity in response to EC antigen presentation (73). Liver sinusoidal ECs can present soluble antigens like carcinoembryonic antigen from colorectal carcinoma together with the co-inhibitory molecule PD-L1, thus promoting tolerance of CD8+ T cells (74). An overexpression of B7-H3 (CD276) on tumor cells has been reported in selected cancers with both stimulatory and inhibitory properties (75). However, B7-H3 expression by the tumor vasculature seems to be a marker of poor prognosis in renal cell carcinoma (76), ovarian carcinomas (77), and endometrial cancers (78). In renal cell carcinoma, an expression of B7-H4, a negative co-stimulatory molecule, has been reported to be associated with cancer progression (79, 80). Expression of B7-H4 by the vasculature was hardly specific to TECs in human renal cell carcinoma compared to adjacent renal tissue vessels (79). A preferential expression of another negative co-inhibitory molecule, TIM-3, has been observed on the endothelium from B-cell lymphoma, with a level of expression closely correlated to dissemination and poor prognosis (81). In addition, FasL expression on TECs could contribute to limit anti-tumoral immunity as it inhibits leukocyte extravasation through apoptosis induction (82). The high levels of endothelial FasL expression in gliomas, together with the inverse correlation observed between FasL expression and CD8+/CD4+ T cells infiltration ratios, indeed suggested that FasL could decrease T-cell infiltration in brain tumors in a subset-selective manner, thus contributing to glioma immune privilege (83).

The decrease of activating co-stimulatory molecules expressed by ECs could be hypothesized but have not been reported. Molecules involved in the direct stimulation of lymphocytes like ICOS-L, CD40, CD80, CD86 are upregulated by angiostatic molecules and Th1 cytokines. An inhibition of these co-stimulatory signals has also been proposed after the identification of markers of tumor endothelium by Seaman et al. (28). Among them, CD137, also called 4-1BB, is a glycoprotein usually expressed by activated T, B and NK cells, DCs, and bone marrow myeloid precursors, and is involved in T-cell co-stimulation. Its expression is undetectable in normal vessels (84) and can be induced by hypoxia (85). TECs seem to specifically express both a membrane-bound form of the molecule and a soluble one (28). Although the exact functions of each of these forms are unclear, soluble CD137 is thought to be antagonistic to the co-stimulatory activity of membrane-bound CD137 on T cells. Thus, sCD137 secreted by TECs may reduce immune activity against tumors. As activation of CD137 on EC induces adhesion molecule expression, like ICAM-1, VCAM-1, and E-selectin (85), high levels of sCD137 could also limit this process.

Another mechanism involving the immunological synapse has been described in tumors. CD31, or platelet endothelial cell adhesion molecule-1 (PECAM-1), can form endothelial junctions and promote leukocyte transendothelial cell migration, respectively (41). But it is also involved in the interactions between T lymphocytes and APC and seems to participate to tumoral immunosuppression. Indeed, a lack of CD31 expression *in vivo* amplified cancer T-cell-mediated rejection, in association with an increase of the threshold of TCR signaling required, leading to a resistance to tolerance induction (86).

#### Soluble and intracellular molecules

Under pro-inflammatory signals, ECs can secrete cytokines and directly activate resting effector cells (34). Some act as mitogens or activators, like IL-1 and IL-15, others influence the differentiation of activated T cells, like IL-6, IL-11, IL-12, and IL-18. GITR-L and IL-6 can also down-regulate Treg activity.

In tumors, immunosuppressive cytokines can be secreted under the tumor microenvironment stimuli. Studies investigating the effect of conditioned media from Lewis lung carcinoma (87) and oral squamous cell carcinoma (88) on ECs observed an increased level of PGE2 and VEGF in the culture supernatants that disrupt NK cell, T-cell, and macrophage functions. High secretions of PGE2, IL-6, TGF-β, and VEGF and a decrease secretion of IL-12 have been observed in ECs isolated from a mouse model of Lewis lung carcinoma (89). Conditioned media from these ECs disrupted T-cell cytokine production in response to anti-CD3 stimulation, and had a decreased ability to activate NK cells and induce macrophage phagocytosis (89). *In vitro*, interactions between microvessel ECs and tumor cells from head and neck squamous cell carcinoma induced the secretion of PGE2 by ECs through an IL-1 pathway (90). Taflin demonstrated in an experimental model of microvascular endothelium that ECs could induce Th17 lymphocytes via IL-6 endothelial synthesis (91). Recently, the prevalence of Th17 cells was found to be elevated in peripheral blood of head and neck squamous cell carcinoma patients. In addition, tumor tissue and tumor-draining lymph nodes were infiltrated by a huge number of Th17 cells representing an important fraction of the TILs (92). Th17 cells are subpopulations of CD4+ T cells favoring the recruitment of neutrophils and the induction of pro-inflammatory cytokines (IL-1, IL-6, TNF-α …) (93). Some of these Th17 cells express immunosuppressive enzymes (CD39, CD73) (94). Their role on the control of tumor is ambivalent, as they can promote the growth of tumor cells via the induction of an inflammatory state in the tumor microenvironment and an increase of angiogenesis (95, 96). But other studies reported an inhibitory role of these cells via the expansion of anti-tumor CD8+ T cells (97).

In DCs, the interaction between CTLA-4 and CD80/CD86 induces indoleamine 2,3-dioxygenase (IDO) expression. This inflammatory enzyme is implicated in the catabolism of the essential amino acid tryptophan and participates in immune tolerance and tumor immunoresistance by simultaneously depleting essential tryptophan and generating immunosuppressive tryptophan metabolites. IDO expression is a mechanism to regulate T-cell activation by APCs and has been considered to be a major mechanism involved in the escape of tumors from the host immune response (98). IDO expression by ECs has been described in tumors but its role is not clearly defined. A study of Batista et al. described endothelial IDO expression specifically in high-grade tumors and not in low-grade (99). However, in renal cell cancer, IDO expression was found nearly specific of ECs from newly formed blood vessels (100) and inversely correlated with the content of proliferating Ki-67+ tumor cells in primary and metastatic clear cell RCC (100). In this tumor, expression of IDO by tumor cells might restrict tumor growth by limiting the influx of tryptophan from the blood to the tumor or generate tumor-toxic metabolites. The role of IDO needs then to be clarified.

## Reversal of Endothelial Barrier: Clinical Applications

Tumor infiltration by lymphocytes depends on cancer cell type and individuals but is a prognostic factor of response to treatment. A recent Nature Cancer Review meta-analysis summarizes the impact of different immune cells on clinical outcome from more than 120 published articles (101). A strong T-cell infiltration associated with good clinical outcome was reported in many different tumors. Establishment of an immunoscore that would include the immune cell density, calculated by numerical quantification of two lymphocyte populations, cytotoxic and memory T cells at the center of the tumor and the invasive margin of tumors, has thus been proposed (102). The level of TILs can also be useful to adapt the treatment. Indeed, in tumors with high levels of TILs, intra-tumoral immunosuppressive mechanisms must be attenuated. In tumors with low levels of TILs, either tumor antigen-presenting process is down-regulated, or the prohibitive tumor endothelial barrier enables T cells to home to tumors, or both. Association between endothelium targeted therapy and immunization boost like vaccination or cell adoptive transfer could enhance anti-tumoral immune response.

### Reversal of immunosuppression by anti-angiogenic therapy

Vascular endothelial growth factor-targeted therapies were initially developed in order to inhibit new blood vessel growth and thus starve tumors of necessary oxygen and nutrients. It has become increasingly apparent, however, that the therapeutic benefit associated with VEGF-targeted therapy was complex, probably involving multiple mechanisms, some of them relying on the improvement of the immune status during tumor development. An increase of B and T cells has been observed in patients with metastatic colorectal cancer treated with bevacizumab, an antibody directed against VEGF (103). This treatment can also promote the differentiation of DCs with a parallel decrease of the immature myeloid cell population in different tumors (104). A decrease of different subsets of immunosuppressive cells, like MDSCs and Tregs, has also been described under sunitinib treatment in metastatic renal cell carcinoma (2, 3), in association with a reversal of type 1 T-cell suppression (3, 105, 106).

The clinical benefit observed on tumor growth made several molecules indicated in first line treatment of renal cell carcinoma and colorectal cancer, in association with chemotherapy. Despite their impact on immunosuppression, no benefit on overall survival has been observed with anti-angiogenic therapies used in monotherapy. These limited results might be explained by the existence of a therapeutic window for their benefit. Indeed, vascular normalization has been observed under anti-angiogenic treatment, with a more mature and functional tumor vasculature able to deliver chemotherapy or immune cells (107–109). However, this effect has been demonstrated to be transient, as prolonged anti-angiogenic treatments finally lead to vasculature rarefaction (110). Combinations between anti-angiogenic therapy and anti-tumoral vaccination are now in clinical development (111) and have already shown promising results in preclinical models (8, 112, 113). A stronger effect of vaccination when associated with sunitinib in a mouse model has been observed, with an increase of CD8+ T-cell infiltration, a decrease in Treg and MDSC infiltration, and a slower tumor growth (113). Bose et al. also demonstrated the benefit of associating vaccination and sunitinib, where the increase of lymphocyte infiltration was associated to vascular normalization and an increase in the expression of adhesion molecules by TECs (114). As anti-angiogenic therapies also modulate peripheral immune populations, the validation of immune parameters as predictive biomarkers of the effect of anti-angiogenic therapy would be of great interest. To face the difficulty to optimize the anti-angiogenic treatment to reach a strong adjuvant effect, tools to induce stable normalization have been proposed, like targeting gene involved in abnormal vascular development like PHD2 (115) or regulator of G-protein signaling 5 (107), or correcting oxygen tension by inositol trispyrophosphate (116).

### Reversal of endothelial cell anergy

Blocking more specifically immune suppressive ECs may help improving the efficacy of existing immunotherapies, particularly those consisting of T cells or NK cells as these cells must pass through the tumor vasculature to infiltrate tumors. Interestingly, EC anergy induced by tumor pro-angiogenic factors (Figure 1) can be reversed under high dose of TNF-α (117). The treatment of mice with NGR–TNF-α, a fusion form of TNF-α with a tumor-homing peptide recognizing specifically TECs, induced intra-tumor upregulation of CAMs, and the infiltration of tumor-specific effector CD8+ T cells. Activation of specific molecules expressed by ECs known to induce immune reactivity has also proved to be beneficial. Use of agonists of CD137, a co-stimulatory molecule identified as a tumor endothelium marker by Seaman et al. (28), or of multivalent RNA aptamers binding CD137 have been shown to enhance anti-tumor CD8 T-cell-mediated immunity in mice (118–120). The therapeutic effects of anti-CD137 agonist antibodies on tumors could be explained by complementary mechanisms, with activation of both immune cells and endothelium. Indeed, they can promote CAMs expression by ECs and then T-cell infiltration, but no effect on angiogenesis or vasculogenesis has been observed (85). Combination of anti-CD137 antibodies with other immunotherapeutic strategies (121–123) and conventional therapies (124) also revealed successful in mouse models.

## Conclusion

Regulation of immunity is one of the numerous functions of endothelium. It is now well-demonstrated that tumor endothelium is implicated in the suppression of adaptive anti-tumoral immune response exerted by the tumor. Tumor endothelium is a prohibitive barrier that inhibits T-cell homing to the tumor and inactivates immune cells through antigen presentation co-inhibitory signals and the expression of immunosuppressive molecules. The immunosuppressive phenotype of TECs is dependent on the tumor microenvironment. The regulation of immunity by tumor vasculature was initially demonstrated by the reversal of immunosuppression observed under anti-angiogenic treatments. Combination between immunotherapies and these treatments reversing TEC phenotype have shown encouraging results promoting the use of these molecules as adjuvant therapies. For that purpose, validation of immune parameters as predictive biomarkers is required. Alternative tools inducing stable normalization are also in development. Further research is needed to identify new endothelial targets and determine how the modulation of EC phenotype could be combined with immunotherapeutic strategies.

## Conflict of Interest Statement

The authors declare that the research was conducted in the absence of any commercial or financial relationships that could be construed as a potential conflict of interest.

## References

[1] Gabrilovich DI Chen HL Girgis KR Cunningham HT Meny GM Nadaf S Production of vascular endothelial growth factor by human tumors inhibits the functional maturation of dendritic cells. Nat Med (1996) 2(10):1096–103 10.1038/nm1096-1096 8837607

[2] Adotevi O Pere H Ravel P Haicheur N Badoual C Merillon N A decrease of regulatory T cells correlates with overall survival after sunitinib-based antiangiogenic therapy in metastatic renal cancer patients. J Immunother (2010) 33(9):991–8 10.1097/CJI.0b013e3181f4c208 20948437

[3] Ko JS Zea AH Rini BI Ireland JL Elson P Cohen P Sunitinib mediates reversal of myeloid-derived suppressor cell accumulation in renal cell carcinoma patients. Clin Cancer Res (2009) 15(6):2148–57 10.1158/1078-0432.CCR-08-1332 19276286

[4] Bruchard M Mignot G Derangere V Chalmin F Chevriaux A Vegran F Chemotherapy-triggered cathepsin B release in myeloid-derived suppressor cells activates the Nlrp3 inflammasome and promotes tumor growth. Nat Med (2013) 19(1):57–64 10.1038/nm.2999 23202296

[5] Chung AS Wu X Zhuang G Ngu H Kasman I Zhang J An interleukin-17-mediated paracrine network promotes tumor resistance to anti-angiogenic therapy. Nat Med (2013) 19(9):1114–23 10.1038/nm.3291 23913124

[6] Hanahan D Weinberg RA Hallmarks of cancer: the next generation. Cell (2011) 144(5):646–74 10.1016/j.cell.2011.02.013 21376230

[7] Motz GT Coukos G Deciphering and reversing tumor immune suppression. Immunity (2013) 39(1):61–73 10.1016/j.immuni.2013.07.005 23890064 PMC3782392

[8] Tartour E Pere H Maillere B Terme M Merillon N Taieb J Angiogenesis and immunity: a bidirectional link potentially relevant for the monitoring of antiangiogenic therapy and the development of novel therapeutic combination with immunotherapy. Cancer Metastasis Rev (2011) 30(1):83–95 10.1007/s10555-011-9281-4 21249423

[9] Facciabene A Motz GT Coukos G T-regulatory cells: key players in tumor immune escape and angiogenesis. Cancer Res (2012) 72(9):2162–71 10.1158/0008-5472.CAN-11-3687 22549946 PMC3342842

[10] Gabrilovich DI Nagaraj S Myeloid-derived suppressor cells as regulators of the immune system. Nat Rev Immunol (2009) 9(3):162–74 10.1038/nri2506 19197294 PMC2828349

[11] Terme M Pernot S Marcheteau E Sandoval F Benhamouda N Colussi O VEGFA-VEGFR pathway blockade inhibits tumor-induced regulatory T-cell proliferation in colorectal cancer. Cancer Res (2013) 73(2):539–49 10.1158/0008-5472.CAN-12-2325 23108136

[12] Terme M Colussi O Marcheteau E Tanchot C Tartour E Taieb J Modulation of immunity by antiangiogenic molecules in cancer. Clin Dev Immunol (2012) 2012:492920 10.1155/2012/492920 23320019 PMC3540780

[13] Curiel TJ Coukos G Zou L Alvarez X Cheng P Mottram P Specific recruitment of regulatory T cells in ovarian carcinoma fosters immune privilege and predicts reduced survival. Nat Med (2004) 10(9):942–9 10.1038/nm1093 15322536

[14] Facciabene A Peng X Hagemann IS Balint K Barchetti A Wang LP Tumour hypoxia promotes tolerance and angiogenesis via CCL28 and T(reg) cells. Nature (2011) 475(7355):226–30 10.1038/nature10169 21753853

[15] Gobert M Treilleux I Bendriss-Vermare N Bachelot T Goddard-Leon S Arfi V Regulatory T cells recruited through CCL22/CCR4 are selectively activated in lymphoid infiltrates surrounding primary breast tumors and lead to an adverse clinical outcome. Cancer Res (2009) 69(5):2000–9 10.1158/0008-5472.CAN-08-2360 19244125

[16] Pere H Montier Y Bayry J Quintin-Colonna F Merillon N Dransart E A CCR4 antagonist combined with vaccines induces antigen-specific CD8+ T cells and tumor immunity against self antigens. Blood (2011) 118(18):4853–62 10.1182/blood-2011-01-329656 21908423

[17] Badoual C Hans S Merillon N Van Ryswick C Ravel P Benhamouda N PD-1-expressing tumor-infiltrating T cells are a favorable prognostic biomarker in HPV-associated head and neck cancer. Cancer Res (2013) 73(1):128–38 10.1158/0008-5472.CAN-12-2606 23135914

[18] Aird WC Spatial and temporal dynamics of the endothelium. J Thromb Haemost (2005) 3(7):1392–406 10.1111/j.1538-7836.2005.01328.x 15892866

[19] Chi JT Chang HY Haraldsen G Jahnsen FL Troyanskaya OG Chang DS Endothelial cell diversity revealed by global expression profiling. Proc Natl Acad Sci U S A (2003) 100(19):10623–8 10.1073/pnas.1434429100 12963823 PMC196854

[20] Aird WC Mechanisms of endothelial cell heterogeneity in health and disease. Circ Res (2006) 98(2):159–62 10.1161/01.RES.0000204553.32549.a7 16456105

[21] Cines DB Pollak ES Buck CA Loscalzo J Zimmerman GA McEver RP Endothelial cells in physiology and in the pathophysiology of vascular disorders. Blood (1998) 91(10):3527–61 9572988

[22] Pober JS Sessa WC Evolving functions of endothelial cells in inflammation. Nat Rev Immunol (2007) 7(10):803–15 10.1038/nri2171 17893694

[23] Grivennikov SI Greten FR Karin M Immunity, inflammation, and cancer. Cell (2010) 140(6):883–99 10.1016/j.cell.2010.01.025 20303878 PMC2866629

[24] de Chaisemartin L Goc J Damotte D Validire P Magdeleinat P Alifano M Characterization of chemokines and adhesion molecules associated with T cell presence in tertiary lymphoid structures in human lung cancer. Cancer Res (2011) 71(20):6391–9 10.1158/0008-5472.CAN-11-0952 21900403

[25] Martinet L Garrido I Filleron T Le Guellec S Bellard E Fournie JJ Human solid tumors contain high endothelial venules: association with T- and B-lymphocyte infiltration and favorable prognosis in breast cancer. Cancer Res (2011) 71(17):5678–87 10.1158/0008-5472.CAN-11-0431 21846823

[26] Di Caro G Bergomas F Grizzi F Doni A Bianchi P Malesci A Occurrence of tertiary lymphoid tissue is associated to T cell infiltration and predicts better prognosis in early stage colorectal cancers. Clin Cancer Res (2014). 10.1158/1078-0432.CCR-13-2590 24523438

[27] Carmeliet P Jain RK Principles and mechanisms of vessel normalization for cancer and other angiogenic diseases. Nat Rev Drug Discov (2011) 10(6):417–27 10.1038/nrd3455 21629292

[28] Seaman S Stevens J Yang MY Logsdon D Graff-Cherry C St Croix B Genes that distinguish physiological and pathological angiogenesis. Cancer Cell (2007) 11(6):539–54 10.1016/j.ccr.2007.04.017 17560335 PMC2039723

[29] St Croix B Rago C Velculescu V Traverso G Romans KE Montgomery E Genes expressed in human tumor endothelium. Science (2000) 289(5482):1197–202 10.1126/science.289.5482.1197 10947988

[30] Madden SL Cook BP Nacht M Weber WD Callahan MR Jiang Y Vascular gene expression in nonneoplastic and malignant brain. Am J Pathol (2004) 165(2):601–8 10.1016/S0002-9440(10)63324-X 15277233 PMC1618572

[31] van Beijnum JR Dings RP van der Linden E Zwaans BM Ramaekers FC Mayo KH Gene expression of tumor angiogenesis dissected: specific targeting of colon cancer angiogenic vasculature. Blood (2006) 108(7):2339–48 10.1182/blood-2006-02-004291 16794251

[32] Buckanovich RJ Sasaroli D O’Brien-Jenkins A Botbyl J Hammond R Katsaros D Tumor vascular proteins as biomarkers in ovarian cancer. J Clin Oncol (2007) 25(7):852–61 10.1200/JCO.2006.08.8583 17327606

[33] Fonsato V Buttiglieri S Deregibus MC Puntorieri V Bussolati B Camussi G Expression of Pax2 in human renal tumor-derived endothelial cells sustains apoptosis resistance and angiogenesis. Am J Pathol (2006) 168(2):706–13 10.2353/ajpath.2006.050776 16436683 PMC1606486

[34] Choi J Enis DR Koh KP Shiao SL Pober JS T lymphocyte-endothelial cell interactions. Annu Rev Immunol (2004) 22:683–709 10.1146/annurev.immunol.22.012703.104639 15032593

[35] Springer TA Traffic signals on endothelium for lymphocyte recirculation and leukocyte emigration. Annu Rev Physiol (1995) 57:827–72 10.1146/annurev.ph.57.030195.004143 7778885

[36] Zitvogel L Tesniere A Kroemer G Cancer despite immunosurveillance: immunoselection and immunosubversion. Nat Rev Immunol (2006) 6(10):715–27 10.1038/nri1936 16977338

[37] Griffioen AW Damen CA Blijham GH Groenewegen G Tumor angiogenesis is accompanied by a decreased inflammatory response of tumor-associated endothelium. Blood (1996) 88(2):667–73 8695814

[38] Kubes P Suzuki M Granger DN Nitric oxide: an endogenous modulator of leukocyte adhesion. Proc Natl Acad Sci U S A (1991) 88(11):4651–5 10.1073/pnas.88.11.4651 1675786 PMC51723

[39] Buckanovich RJ Facciabene A Kim S Benencia F Sasaroli D Balint K Endothelin B receptor mediates the endothelial barrier to T cell homing to tumors and disables immune therapy. Nat Med (2008) 14(1):28–36 10.1038/nm1699 18157142

[40] Bouzin C Brouet A De Vriese J Dewever J Feron O Effects of vascular endothelial growth factor on the lymphocyte-endothelium interactions: identification of caveolin-1 and nitric oxide as control points of endothelial cell anergy. J Immunol (2007) 178(3):1505–11 17237399 10.4049/jimmunol.178.3.1505

[41] Muller WA Mechanisms of leukocyte transendothelial migration. Annu Rev Pathol (2011) 6:323–44 10.1146/annurev-pathol-011110-130224 21073340 PMC3628537

[42] Soncin F Mattot V Lionneton F Spruyt N Lepretre F Begue A VE-statin, an endothelial repressor of smooth muscle cell migration. EMBO J (2003) 22(21):5700–11 10.1093/emboj/cdg549 14592969 PMC275406

[43] Parker LH Schmidt M Jin SW Gray AM Beis D Pham T The endothelial-cell-derived secreted factor Egfl7 regulates vascular tube formation. Nature (2004) 428(6984):754–8 10.1038/nature02416 15085134

[44] Delfortrie S Pinte S Mattot V Samson C Villain G Caetano B Egfl7 promotes tumor escape from immunity by repressing endothelial cell activation. Cancer Res (2011) 71(23):7176–86 10.1158/0008-5472.CAN-11-1301 22037871

[45] Wu F Yang LY Li YF Ou DP Chen DP Fan C Novel role for epidermal growth factor-like domain 7 in metastasis of human hepatocellular carcinoma. Hepatology (2009) 50(6):1839–50 10.1002/hep.23197 19824075

[46] Huang CH Li XJ Zhou YZ Luo Y Li C Yuan XR Expression and clinical significance of EGFL7 in malignant glioma. J Cancer Res Clin Oncol (2010) 136(11):1737–43 10.1007/s00432-010-0832-9 20213100 PMC11828204

[47] Nelson J Bagnato A Battistini B Nisen P The endothelin axis: emerging role in cancer. Nat Rev Cancer (2003) 3(2):110–6 10.1038/nrc990 12563310

[48] Matsuura A Yamochi W Hirata K Kawashima S Yokoyama M Stimulatory interaction between vascular endothelial growth factor and endothelin-1 on each gene expression. Hypertension (1998) 32(1):89–95 10.1161/01.HYP.32.1.89 9674643

[49] Salani D Taraboletti G Rosano L Di Castro V Borsotti P Giavazzi R Endothelin-1 induces an angiogenic phenotype in cultured endothelial cells and stimulates neovascularization in vivo. Am J Pathol (2000) 157(5):1703–11 10.1016/S0002-9440(10)64807-9 11073829 PMC1885730

[50] Spinella F Rosano L Di Castro V Natali PG Bagnato A Endothelin-1 induces vascular endothelial growth factor by increasing hypoxia-inducible factor-1alpha in ovarian carcinoma cells. J Biol Chem (2002) 277(31):27850–5 10.1074/jbc.M202421200 12023962

[51] Wu MH Huang CY Lin JA Wang SW Peng CY Cheng HC Endothelin-1 promotes vascular endothelial growth factor-dependent angiogenesis in human chondrosarcoma cells. Oncogene (2013). 10.1038/onc.2013.109 23584483

[52] Bernabeu C Lopez-Novoa JM Quintanilla M The emerging role of TGF-beta superfamily coreceptors in cancer. Biochim Biophys Acta (2009) 1792(10):954–73 10.1016/j.bbadis.2009.07.003 19607914

[53] Shovlin CL Hereditary haemorrhagic telangiectasia: pathophysiology, diagnosis and treatment. Blood Rev (2010) 24(6):203–19 10.1016/j.blre.2010.07.001 20870325

[54] Rossi E Sanz-Rodriguez F Eleno N Duwell A Blanco FJ Langa C Endothelial endoglin is involved in inflammation: role in leukocyte adhesion and transmigration. Blood (2013) 121(2):403–15 10.1182/blood-2012-06-435347 23074273

[55] Takahashi N Kawanishi-Tabata R Haba A Tabata M Haruta Y Tsai H Association of serum endoglin with metastasis in patients with colorectal, breast, and other solid tumors, and suppressive effect of chemotherapy on the serum endoglin. Clin Cancer Res (2001) 7(3):524–32 11297243

[56] Li C Guo B Wilson PB Stewart A Byrne G Bundred N Plasma levels of soluble CD105 correlate with metastasis in patients with breast cancer. Int J Cancer (2000) 89(2):122–6 10.1002/(SICI)1097-0215(20000320)89:2<122::AID-IJC4>3.0.CO;2-M 10754488

[57] Zheng C Qiu Y Zeng Q Zhang Y Lu D Yang D Endothelial CD146 is required for in vitro tumor-induced angiogenesis: the role of a disulfide bond in signaling and dimerization. Int J Biochem Cell Biol (2009) 41(11):2163–72 10.1016/j.biocel.2009.03.014 19782948

[58] Harhouri K Kebir A Guillet B Foucault-Bertaud A Voytenko S Piercecchi-Marti MD Soluble CD146 displays angiogenic properties and promotes neovascularization in experimental hind-limb ischemia. Blood (2010) 115(18):3843–51 10.1182/blood-2009-06-229591 20185588

[59] Bardin N Blot-Chabaud M Despoix N Kebir A Harhouri K Arsanto JP CD146 and its soluble form regulate monocyte transendothelial migration. Arterioscler Thromb Vasc Biol (2009) 29(5):746–53 10.1161/ATVBAHA.108.183251 19229070

[60] Guezguez B Vigneron P Lamerant N Kieda C Jaffredo T Dunon D Dual role of melanoma cell adhesion molecule (MCAM)/CD146 in lymphocyte endothelium interaction: MCAM/CD146 promotes rolling via microvilli induction in lymphocyte and is an endothelial adhesion receptor. J Immunol (2007) 179(10):6673–85 17982057 10.4049/jimmunol.179.10.6673

[61] Yan X Lin Y Yang D Shen Y Yuan M Zhang Z A novel anti-CD146 monoclonal antibody, AA98, inhibits angiogenesis and tumor growth. Blood (2003) 102(1):184–91 10.1182/blood-2002-04-1004 12609848

[62] Shetty S Weston CJ Oo YH Westerlund N Stamataki Z Youster J Common lymphatic endothelial and vascular endothelial receptor-1 mediates the transmigration of regulatory T cells across human hepatic sinusoidal endothelium. J Immunol (2011) 186(7):4147–55 10.4049/jimmunol.1002961 21368224 PMC6016742

[63] Nummer D Suri-Payer E Schmitz-Winnenthal H Bonertz A Galindo L Antolovich D Role of tumor endothelium in CD4+ CD25+ regulatory T cell infiltration of human pancreatic carcinoma. J Natl Cancer Inst (2007) 99(15):1188–99 10.1093/jnci/djm064 17652277

[64] Pober JS Immunobiology of human vascular endothelium. Immunol Res (1999) 19(2–3):225–32 10.1007/BF02786490 10493176

[65] Pober JS Kluger MS Schechner JS Human endothelial cell presentation of antigen and the homing of memory/effector T cells to skin. Ann N Y Acad Sci (2001) 941:12–25 10.1111/j.1749-6632.2001.tb03706.x 11594565

[66] Benlalam H Jalil A Hasmim M Pang B Tamouza R Mitterrand M Gap junction communication between autologous endothelial and tumor cells induce cross-recognition and elimination by specific CTL. J Immunol (2009) 182(5):2654–64 10.4049/jimmunol.0800815 19234159

[67] Lohse AW Knolle PA Bilo K Uhrig A Waldmann C Ibe M Antigen-presenting function and B7 expression of murine sinusoidal endothelial cells and Kupffer cells. Gastroenterology (1996) 110(4):1175–81 10.1053/gast.1996.v110.pm8613007 8613007

[68] Diehl L Schurich A Grochtmann R Hegenbarth S Chen L Knolle PA Tolerogenic maturation of liver sinusoidal endothelial cells promotes B7-homolog 1-dependent CD8+ T cell tolerance. Hepatology (2008) 47(1):296–305 10.1002/hep.21965 17975811

[69] Arteta B Lasuen N Lopategi A Sveinbjornsson B Smedsrod B Vidal-Vanaclocha F Colon carcinoma cell interaction with liver sinusoidal endothelium inhibits organ-specific antitumor immunity through interleukin-1-induced mannose receptor in mice. Hepatology (2010) 51(6):2172–82 10.1002/hep.23590 20513002

[70] Pober JS Tellides G Participation of blood vessel cells in human adaptive immune responses. Trends Immunol (2012) 33(1):49–57 10.1016/j.it.2011.09.006 22030237 PMC3253953

[71] Hughes CC Savage CO Pober JS Endothelial cells augment T cell interleukin 2 production by a contact-dependent mechanism involving CD2/LFA-3 interaction. J Exp Med (1990) 171(5):1453–67 10.1084/jem.171.5.1453 1692079 PMC2187887

[72] Eppihimer MJ Gunn J Freeman GJ Greenfield EA Chernova T Erickson J Expression and regulation of the PD-L1 immunoinhibitory molecule on microvascular endothelial cells. Microcirculation (2002) 9(2):133–45 10.1080/713774061 11932780 PMC3740166

[73] Rodig N Ryan T Allen JA Pang H Grabie N Chernova T Endothelial expression of PD-L1 and PD-L2 down-regulates CD8+ T cell activation and cytolysis. Eur J Immunol (2003) 33(11):3117–26 10.1002/eji.200324270 14579280

[74] Hochst B Schildberg FA Bottcher J Metzger C Huss S Turler A Liver sinusoidal endothelial cells contribute to CD8 T cell tolerance toward circulating carcinoembryonic antigen in mice. Hepatology (2012) 56(5):1924–33 10.1002/hep.25844 22610745

[75] Loos M Hedderich DM Friess H Kleeff J B7-h3 and its role in antitumor immunity. Clin Dev Immunol (2010) 2010:683875 10.1155/2010/683875 21127709 PMC2993024

[76] Crispen PL Sheinin Y Roth TJ Lohse CM Kuntz SM Frigola X Tumor cell and tumor vasculature expression of B7-H3 predict survival in clear cell renal cell carcinoma. Clin Cancer Res (2008) 14(16):5150–7 10.1158/1078-0432.CCR-08-0536 18694993 PMC2789387

[77] Zang X Sullivan PS Soslow RA Waitz R Reuter VE Wilton A Tumor associated endothelial expression of B7-H3 predicts survival in ovarian carcinomas. Mod Pathol (2010) 23(8):1104–12 10.1038/modpathol.2010.95 20495537 PMC2976590

[78] Brunner A Hinterholzer S Riss P Heinze G Brustmann H Immunoexpression of B7-H3 in endometrial cancer: relation to tumor T-cell infiltration and prognosis. Gynecol Oncol (2012) 124(1):105–11 10.1016/j.ygyno.2011.09.012 21982044

[79] Krambeck AE Thompson RH Dong H Lohse CM Park ES Kuntz SM B7-H4 expression in renal cell carcinoma and tumor vasculature: associations with cancer progression and survival. Proc Natl Acad Sci U S A (2006) 103(27):10391–6 10.1073/pnas.0600937103 16798883 PMC1502468

[80] Thompson RH Gillett MD Cheville JC Lohse CM Dong H Webster WS Costimulatory B7-H1 in renal cell carcinoma patients: indicator of tumor aggressiveness and potential therapeutic target. Proc Natl Acad Sci U S A (2004) 101(49):17174–9 10.1073/pnas.0406351101 15569934 PMC534606

[81] Huang X Bai X Cao Y Wu J Huang M Tang D Lymphoma endothelium preferentially expresses Tim-3 and facilitates the progression of lymphoma by mediating immune evasion. J Exp Med (2010) 207(3):505–20 10.1084/jem.20090397 20176801 PMC2839144

[82] Sata M Walsh K TNFalpha regulation of Fas ligand expression on the vascular endothelium modulates leukocyte extravasation. Nat Med (1998) 4(4):415–20 10.1038/nm0498-415 9546786 PMC2828686

[83] Yu JS Lee PK Ehtesham M Samoto K Black KL Wheeler CJ Intratumoral T cell subset ratios and Fas ligand expression on brain tumor endothelium. J Neurooncol (2003) 64(1–2):55–61 10.1023/A:1024933925645 12952286

[84] Broll K Richter G Pauly S Hofstaedter F Schwarz H CD137 expression in tumor vessel walls. High correlation with malignant tumors. Am J Clin Pathol (2001) 115(4):543–9 10.1309/E343-KMYX-W3Y2-10KY 11293902

[85] Palazon A Teijeira A Martinez-Forero I Hervas-Stubbs S Roncal C Penuelas I Agonist anti-CD137 mAb act on tumor endothelial cells to enhance recruitment of activated T lymphocytes. Cancer Res (2011) 71(3):801–11 10.1158/0008-5472.CAN-10-1733 21266358

[86] Ma L Mauro C Cornish GH Chai JG Coe D Fu H Ig gene-like molecule CD31 plays a nonredundant role in the regulation of T-cell immunity and tolerance. Proc Natl Acad Sci U S A (2010) 107(45):19461–6 10.1073/pnas.1011748107 20978210 PMC2984185

[87] Mulligan JK Lathers DM Young MR Tumors skew endothelial cells to disrupt NK cell, T-cell and macrophage functions. Cancer Immunol Immunother (2008) 57(7):951–61 10.1007/s00262-007-0425-x 18058097 PMC3333838

[88] Mulligan JK Day TA Gillespie MB Rosenzweig SA Young MR Secretion of vascular endothelial growth factor by oral squamous cell carcinoma cells skews endothelial cells to suppress T-cell functions. Hum Immunol (2009) 70(6):375–82 10.1016/j.humimm.2009.01.014 19480853 PMC2746465

[89] Mulligan JK Young MR Tumors induce the formation of suppressor endothelial cells in vivo. Cancer Immunol Immunother (2010) 59(2):267–77 10.1007/s00262-009-0747-y 19669642 PMC3337521

[90] Casos K Siguero L Fernandez-Figueras MT Leon X Sarda MP Vila L Tumor cells induce COX-2 and mPGES-1 expression in microvascular endothelial cells mainly by means of IL-1 receptor activation. Microvasc Res (2011) 81(3):261–8 10.1016/j.mvr.2011.01.006 21277871

[91] Taflin C Favier B Baudhuin J Savenay A Hemon P Bensussan A Human endothelial cells generate Th17 and regulatory T cells under inflammatory conditions. Proc Natl Acad Sci U S A (2011) 108(7):2891–6 10.1073/pnas.1011811108 21282653 PMC3041137

[92] Kesselring R Thiel A Pries R Trenkle T Wollenberg B Human Th17 cells can be induced through head and neck cancer and have a functional impact on HNSCC development. Br J Cancer (2010) 103(8):1245–54 10.1038/sj.bjc.6605891 20877351 PMC2967064

[93] Wilke CM Bishop K Fox D Zou W Deciphering the role of Th17 cells in human disease. Trends Immunol (2011) 32(12):603–11 10.1016/j.it.2011.08.003 21958759 PMC3224806

[94] Chalmin F Mignot G Bruchard M Chevriaux A Vegran F Hichami A Stat3 and Gfi-1 transcription factors control Th17 cell immunosuppressive activity via the regulation of ectonucleotidase expression. Immunity (2012) 36(3):362–73 10.1016/j.immuni.2011.12.019 22406269

[95] Tartour E Fossiez F Joyeux I Galinha A Gey A Claret E Interleukin 17, a T-cell-derived cytokine, promotes tumorigenicity of human cervical tumors in nude mice. Cancer Res (1999) 59(15):3698–704 10446984

[96] Numasaki M Fukushi J Ono M Narula SK Zavodny PJ Kudo T Interleukin-17 promotes angiogenesis and tumor growth. Blood (2003) 101(7):2620–7 10.1182/blood-2002-05-1461 12411307

[97] Benchetrit F Ciree A Vives V Warnier G Gey A Sautes-Fridman C Interleukin-17 inhibits tumor cell growth by means of a T-cell-dependent mechanism. Blood (2002) 99(6):2114–21 10.1182/blood.V99.6.2114 11877287

[98] Munn DH Mellor AL Indoleamine 2,3-dioxygenase and tumor-induced tolerance. J Clin Invest (2007) 117(5):1147–54 10.1172/JCI31178 17476344 PMC1857253

[99] Batista CE Juhasz C Muzik O Kupsky WJ Barger G Chugani HT Imaging correlates of differential expression of indoleamine 2,3-dioxygenase in human brain tumors. Mol Imaging Biol (2009) 11(6):460–6 10.1007/s11307-009-0225-0 19434461 PMC2763988

[100] Riesenberg R Weiler C Spring O Eder M Buchner A Popp T Expression of indoleamine 2,3-dioxygenase in tumor endothelial cells correlates with long-term survival of patients with renal cell carcinoma. Clin Cancer Res (2007) 13(23):6993–7002 10.1158/1078-0432.CCR-07-0942 18056175

[101] Fridman WH Pages F Sautes-Fridman C Galon J The immune contexture in human tumours: impact on clinical outcome. Nat Rev Cancer (2012) 12(4):298–306 10.1038/nrc3245 22419253

[102] Galon J Pages F Marincola FM Thurin M Trinchieri G Fox BA The immune score as a new possible approach for the classification of cancer. J Transl Med (2012) 10:1 10.1186/1479-5876-10-1 22214470 PMC3269368

[103] Manzoni M Rovati B Ronzoni M Loupakis F Mariucci S Ricci V Immunological effects of bevacizumab-based treatment in metastatic colorectal cancer. Oncology (2010) 79(3–4):187–96 10.1159/000320609 21358206

[104] Osada T Chong G Tansik R Hong T Spector N Kumar R The effect of anti-VEGF therapy on immature myeloid cell and dendritic cells in cancer patients. Cancer Immunol Immunother (2008) 57(8):1115–24 10.1007/s00262-007-0441-x 18193223 PMC4110970

[105] Finke JH Rini B Ireland J Rayman P Richmond A Golshayan A Sunitinib reverses type-1 immune suppression and decreases T-regulatory cells in renal cell carcinoma patients. Clin Cancer Res (2008) 14(20):6674–82 10.1158/1078-0432.CCR-07-5212 18927310

[106] Garcia JA Mekhail T Elson P Triozzi P Nemec C Dreicer R Clinical and immunomodulatory effects of bevacizumab and low-dose interleukin-2 in patients with metastatic renal cell carcinoma: results from a phase II trial. BJU Int (2010) 107(4):562–70 10.1111/j.1464-410X.2010.09573.x 20840548

[107] Hamzah J Jugold M Kiessling F Rigby P Manzur M Marti HH Vascular normalization in Rgs5-deficient tumours promotes immune destruction. Nature (2008) 453(7193):410–4 10.1038/nature06868 18418378

[108] Shrimali RK Yu Z Theoret MR Chinnasamy D Restifo NP Rosenberg SA Antiangiogenic agents can increase lymphocyte infiltration into tumor and enhance the effectiveness of adoptive immunotherapy of cancer. Cancer Res (2010) 70(15):6171–80 10.1158/0008-5472.CAN-10-0153 20631075 PMC2912959

[109] Li B Lalani AS Harding TC Luan B Koprivnikar K Huan Tu G Vascular endothelial growth factor blockade reduces intratumoral regulatory T cells and enhances the efficacy of a GM-CSF-secreting cancer immunotherapy. Clin Cancer Res (2006) 12(22):6808–16 10.1158/1078-0432.CCR-06-1558 17121902

[110] Winkler F Kozin SV Tong RT Chae SS Booth MF Garkavtsev I Kinetics of vascular normalization by VEGFR2 blockade governs brain tumor response to radiation: role of oxygenation, angiopoietin-1, and matrix metalloproteinases. Cancer Cell (2004) 6(6):553–63 10.1016/j.ccr.2004.10.011 15607960

[111] Chi N Maranchie JK Appleman LJ Storkus WJ Update on vaccine development for renal cell cancer. Open Access J Urol (2010) 2:125–41 24198621 10.2147/RRU.S7242 PMC3703676

[112] Jaini R Rayman P Cohen PA Finke JH Tuohy VK Combination of sunitinib with anti-tumor vaccination inhibits T cell priming and requires careful scheduling to achieve productive immunotherapy. Int J Cancer (2014) 134(7):1695–705 10.1002/ijc.28488 24105638 PMC3947113

[113] Farsaci B Higgins JP Hodge JW Consequence of dose scheduling of sunitinib on host immune response elements and vaccine combination therapy. Int J Cancer (2012) 130(8):1948–59 10.1002/ijc.26219 21633954 PMC3232304

[114] Bose A Taylor JL Alber S Watkins SC Garcia JA Rini BI Sunitinib facilitates the activation and recruitment of therapeutic anti-tumor immunity in concert with specific vaccination. Int J Cancer (2011) 129(9):2158–70 10.1002/ijc.25863 21170961 PMC3110980

[115] Mazzone M Dettori D Leite de Oliveira R Loges S Schmidt T Jonckx B Heterozygous deficiency of PHD2 restores tumor oxygenation and inhibits metastasis via endothelial normalization. Cell (2009) 136(5):839–51 10.1016/j.cell.2009.01.020 19217150 PMC4037868

[116] Kieda C El Hafny-Rahbi B Collet G Lamerant-Fayel N Grillon C Guichard A Stable tumor vessel normalization with pO(2) increase and endothelial PTEN activation by inositol trispyrophosphate brings novel tumor treatment. J Mol Med (Berl) (2013) 91(7):883–99 10.1007/s00109-013-0992-6 23471434 PMC3695680

[117] Calcinotto A Grioni M Jachetti E Curnis F Mondino A Parmiani G Targeting TNF-alpha to neoangiogenic vessels enhances lymphocyte infiltration in tumors and increases the therapeutic potential of immunotherapy. J Immunol (2012) 188(6):2687–94 10.4049/jimmunol.1101877 22323546

[118] Mittler RS Foell J McCausland M Strahotin S Niu L Bapat A Anti-CD137 antibodies in the treatment of autoimmune disease and cancer. Immunol Res (2004) 29(1–3):197–208 10.1385/IR:29:1-3:197 15181282

[119] Melero I Shuford WW Newby SA Aruffo A Ledbetter JA Hellstrom KE Monoclonal antibodies against the 4-1BB T-cell activation molecule eradicate established tumors. Nat Med (1997) 3(6):682–5 10.1038/nm0697-682 9176498

[120] Hernandez-Chacon JA Li Y Wu RC Bernatchez C Wang Y Weber JS Costimulation through the CD137/4-1BB pathway protects human melanoma tumor-infiltrating lymphocytes from activation-induced cell death and enhances antitumor effector function. J Immunother (2011) 34(3):236–50 10.1097/CJI.0b013e318209e7ec 21389874 PMC3063939

[121] Ito F Li Q Shreiner AB Okuyama R Jure-Kunkel MN Teitz-Tennenbaum S Anti-CD137 monoclonal antibody administration augments the antitumor efficacy of dendritic cell-based vaccines. Cancer Res (2004) 64(22):8411–9 10.1158/0008-5472.CAN-04-0590 15548712

[122] Tirapu I Arina A Mazzolini G Duarte M Alfaro C Feijoo E Improving efficacy of interleukin-12-transfected dendritic cells injected into murine colon cancer with anti-CD137 monoclonal antibodies and alloantigens. Int J Cancer (2004) 110(1):51–60 10.1002/ijc.20093 15054868

[123] Kwong B Gai SA Elkhader J Wittrup KD Irvine DJ Localized immunotherapy via liposome-anchored anti-CD137 + IL-2 prevents lethal toxicity and elicits local and systemic antitumor immunity. Cancer Res (2013) 73(5):1547–58 10.1158/0008-5472.CAN-12-3343 23436794 PMC3594475

[124] Kim YH Choi BK Kim KH Kang SW Kwon BS Combination therapy with cisplatin and anti-4-1BB: synergistic anticancer effects and amelioration of cisplatin-induced nephrotoxicity. Cancer Res (2008) 68(18):7264–9 10.1158/0008-5472.CAN-08-1365 18794112 PMC2551756

